# Methyl 2-(2-bromo­benzyl­idene)-5-(4-hy­droxy­phen­yl)-7-methyl-3-oxo-2,3-dihydro-5*H*-1,3-thia­zolo[3,2-*a*]pyrimidine-6-carboxyl­ate

**DOI:** 10.1107/S1600536812013311

**Published:** 2012-03-31

**Authors:** H. Nagarajaiah, Nikhath Fathima, Noor Shahina Begum

**Affiliations:** a Department of Studies in Chemistry, Bangalore University, Bangalore 560 001, Karnataka, India

## Abstract

In the title compound, C_22_H_17_BrN_2_O_4_S, the central dihydropyrimidine ring, with a chiral C atom, is significantly puckered and adopts a half-chair conformation with the chiral C atom displaced from the mean plane of the remaining ring atoms by 0.305 (6) Å. The hydroxy-phenyl ring is positioned axially to the pyrimidine ring and almost bisects it, the dihedral angle between the mean-planes of the two rings being 89.78 (12)°. The meth­oxy­carbonyl group is disordered over two sites with an occupancy ratio of 0.568 (5):0.432 (5), resulting in a major and a minor conformer. In the crystal, O—H⋯N and C—H⋯S inter­actions result in sheets along the *c* axis. The supra­molecular assembly is stabilized by π–π stacking inter­actions between the 2-bromo­benzyl­idene and thia­zolopyrimidine rings [centroid–centroid distance = 3.632 (1) Å]. In addition, C—H⋯π inter­actions are also observed in the crystal structure.

## Related literature

For therapeutic and medicinal properties of thia­zolopyrimidine derivatives, see: Kappe (2000 ▶); Ozair *et al.* (2010 ▶). For a related structure, see: Nagarajaiah & Begum (2011 ▶).
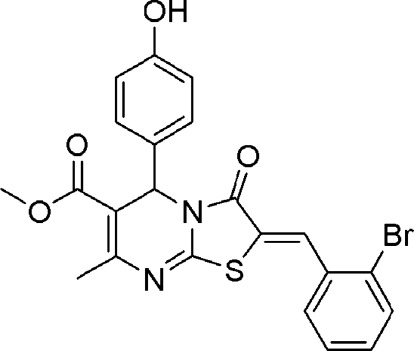


## Experimental

### 

#### Crystal data

C_22_H_17_BrN_2_O_4_S
*M* *_r_* = 485.35
Monoclinic, 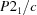

*a* = 9.851 (2) Å
*b* = 23.461 (6) Å
*c* = 9.416 (2) Å
β = 111.229 (5)°
*V* = 2028.5 (8) Å^3^
*Z* = 4
Mo *K*α radiation
μ = 2.16 mm^−1^
*T* = 296 K
0.18 × 0.16 × 0.16 mm

#### Data collection

Bruker SMART APEX CCD detector diffractometer
Absorption correction: multi-scan (*SADABS*, Bruker, 1998 ▶) *T*_min_ = 0.697, *T*_max_ = 0.724
12267 measured reflections
4409 independent reflections
2563 reflections with *I* > 2σ(*I*)
*R*_int_ = 0.054

#### Refinement

*R*[*F* ^2^ > 2σ(*F* ^2^)] = 0.059
*wR*(*F* ^2^) = 0.178
*S* = 1.04
4409 reflections
286 parameters
H-atom parameters constrained
Δρ_max_ = 1.04 e Å^−3^
Δρ_min_ = −0.87 e Å^−3^

### 

Data collection: *SMART* (Bruker, 1998 ▶); cell refinement: *SAINT-Plus* (Bruker, 1998 ▶); data reduction: *SAINT-Plus*; program(s) used to solve structure: *SHELXS97* (Sheldrick, 2008 ▶); program(s) used to refine structure: *SHELXL97* (Sheldrick, 2008 ▶); molecular graphics: *ORTEP-3* (Farrugia, 1997 ▶) and *CAMERON* (Watkin *et al.*, 1996 ▶); software used to prepare material for publication: *WinGX* (Farrugia, 1999 ▶).

## Supplementary Material

Crystal structure: contains datablock(s) global, I. DOI: 10.1107/S1600536812013311/pv2521sup1.cif

Structure factors: contains datablock(s) I. DOI: 10.1107/S1600536812013311/pv2521Isup2.hkl

Supplementary material file. DOI: 10.1107/S1600536812013311/pv2521Isup3.cml

Additional supplementary materials:  crystallographic information; 3D view; checkCIF report

## Figures and Tables

**Table 1:** Hydrogen-bond geometry (Å, °) *Cg*1 is the centroid of the C10–C15 benzene ring.

*D*—H⋯*A*	*D*—H	H⋯*A*	*D*⋯*A*	*D*—H⋯*A*
O4—H4⋯N2^i^	0.82	1.96	2.782 (4)	178
C4*B*—H4*B*1⋯S1^ii^	0.96	2.80	3.621 (12)	144
C1—H1*C*⋯*Cg*1^iii^	0.96	2.78	3.585 (5)	142

Symmetry codes: (i) 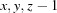; (ii) 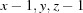; (iii) 
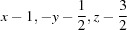
.

**Fig. 1.**
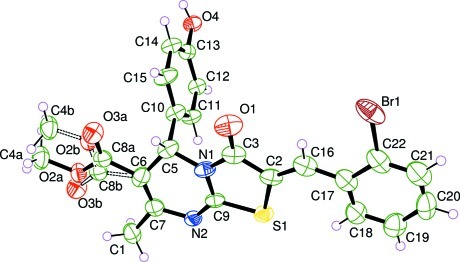
The molecular structure of the title compound with the atom numbering scheme. Displacement ellipsoids are drawn at the 50% probability level. H atoms are presented as small spheres of arbitrary radius.

**Fig. 2.**
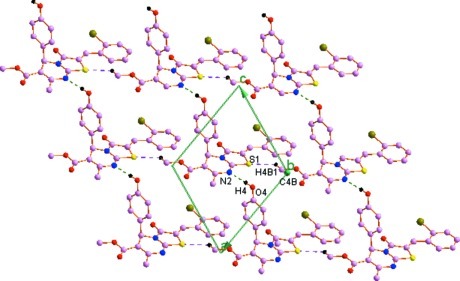
A view of the intermolecular hydrogen bonds (dotted lines) in the crystal structure of the title compound. H atoms non-participating in hydrogen-bonding were omitted for clarity.

## supplementary crystallographic information

### Comment

The title compound is a representative of thiazolopyrimidine derivatives, which have recently emerged as target molecules due to their therapeutic and medicinal properties (Kappe, 2000) such as anti-inflammatory and antinociceptive activities (Ozair *et al.* 2010) in addition to being calcium channel blockers.

In the title molecule (Fig. 1), the 4-hydroxy-phenyl group adopts a psuedo synperiplanar conformation with respect to C5—H5 bond. The central pyrimidine ring with a chiral C5 atom is significantly puckered and adopts a half chair conformation with C5 displaced from the mean plane of the remaining ring atoms (C6/C7/C9//N2/N1) by 0.305 (6) Å. The hydroxy-phenyl ring is positioned axially to the pyrimidine ring and almost bisects it with a dihedral angle between the mean-planes of the two rings being 89.78 (12)°. The methoxycarbonyl group in the title compound is disordered in which the carbon atoms C8, C4 and the oxygen atoms O2 and O3 are located over two sites (C8A/C8B,C4A/C4B, O2A/O2B and O3A/O3B) with site occupancy ratio 0.568 (5):0.432 (5) resulting in a major and a minor conformers. The crystal structure is primarily stabilized by intermolecular O4—H4···N2 and C4B—H4B1···S1 interactions which result in two dimensional sheets along the *c*-axis (Fig. 2). The molecular packing is further stabilized by π—π stacking interactions between the thiazolopyrimidine and 2-bromo-benzylidene rings. The C3···C21 (*x* - 1, *y*, *z* - 1) disposed at a distance of 3.632 (1) Å. In addition C1—H1···*Cg*1 interactions (*Cg*1 being the centroid of the benzene ring C10–C15, Table 1) are also observed. The bond lengths and angles in the title molecule are in close agreement with the corresponding bond lengths and angles reported in a similar compound (Nagarajaiah & Begum, 2011).

### Experimental

A mixture of 4-(4-hydroxy-phenyl)-6-methyl-2-thioxo-1,2,3, 4-tetrahydro-pyrimidine-5-carboxylic acid methyl ester (0.01 mol), chloroaceticacid (0.01 mol), 2-bromo benzaldehyde (0.01 mol) and sodium acetate (1.5 g) was taken in a mixture of glacial acetic acid and acetic anhydride (25 ml; 1:1) and refluxed for 10 hr. The reaction mixture was concentrated and the solid thus obtained was filtered and recrystallized with ethyl acetate to get the title compound (yield = 78%, m.p. 468–470 K). The compound was recrystallized by slow evaporation of an ethyl acetate-ethanol (6:4) solution, yielding pale yellow single crystals suitable for X-ray diffraction.

### Refinement

The H atoms were placed at calculated positions in the riding model approximation with O—H = 0.82 Å and C—H = 0.93, 0.96 and 0.98 Å for aryl, methyl and methyne H-atoms respectively, with *U*_iso_(H) = 1.5*U*_eq_(C) for methyl H atoms and 1.2*U*_eq_(C/O) for other H atom.

### Crystal data

**Table:**

C_22_H_17_BrN_2_O_4_S	*F*(000) = 984
*M**_r_* = 485.35	*D*_x_ = 1.589 Mg m^−^^3^
Monoclinic, *P*2_1_/*c*	Mo *K*α radiation, λ = 0.71073 Å
Hall symbol: -P 2ybc	Cell parameters from 4409 reflections
*a* = 9.851 (2) Å	θ = 2.2–27.0°
*b* = 23.461 (6) Å	µ = 2.16 mm^−^^1^
*c* = 9.416 (2) Å	*T* = 296 K
β = 111.229 (5)°	Block, yellow
*V* = 2028.5 (8) Å^3^	0.18 × 0.16 × 0.16 mm
*Z* = 4	

### Data collection

**Table:**

Bruker SMART APEX CCD detector diffractometer	4409 independent reflections
Radiation source: fine-focus sealed tube	2563 reflections with *I* > 2σ(*I*)
Graphite monochromator	*R*_int_ = 0.054
ω scans	θ_max_ = 27.0°, θ_min_ = 2.2°
Absorption correction: multi-scan (*SADABS*, Bruker, 1998)	*h* = −12→7
*T*_min_ = 0.697, *T*_max_ = 0.724	*k* = −28→29
12267 measured reflections	*l* = −11→12

### Refinement

**Table:**

Refinement on *F*^2^	Primary atom site location: structure-invariant direct methods
Least-squares matrix: full	Secondary atom site location: difference Fourier map
*R*[*F*^2^ > 2σ(*F*^2^)] = 0.059	Hydrogen site location: inferred from neighbouring sites
*wR*(*F*^2^) = 0.178	H-atom parameters constrained
*S* = 1.04	*w* = 1/[σ^2^(*F*_o_^2^) + (0.0878*P*)^2^] where *P* = (*F*_o_^2^ + 2*F*_c_^2^)/3
4409 reflections	(Δ/σ)_max_ < 0.001
286 parameters	Δρ_max_ = 1.04 e Å^−^^3^
0 restraints	Δρ_min_ = −0.87 e Å^−^^3^

### Special details

**Table:**

Geometry. All e.s.d.'s (except the e.s.d. in the dihedral angle between two l.s. planes) are estimated using the full covariance matrix. The cell e.s.d.'s are taken into account individually in the estimation of e.s.d.'s in distances, angles and torsion angles; correlations between e.s.d.'s in cell parameters are only used when they are defined by crystal symmetry. An approximate (isotropic) treatment of cell e.s.d.'s is used for estimating e.s.d.'s involving l.s. planes.
Refinement. Refinement of *F*^2^ against ALL reflections. The weighted *R*-factor *wR* and goodness of fit *S* are based on *F*^2^, conventional *R*-factors *R* are based on *F*, with *F* set to zero for negative *F*^2^. The threshold expression of *F*^2^ > 2σ(*F*^2^) is used only for calculating *R*-factors(gt) *etc*. and is not relevant to the choice of reflections for refinement. *R*-factors based on *F*^2^ are statistically about twice as large as those based on *F*, and *R*- factors based on ALL data will be even larger.

### Fractional atomic coordinates and isotropic or equivalent isotropic displacement parameters (Å^2^)

**Table:**

	*x*	*y*	*z*	*U*_iso_*/*U*_eq_	Occ. (<1)
Br1	0.94209 (7)	0.12175 (2)	0.43004 (8)	0.0693 (3)	
S1	0.69429 (13)	−0.08352 (5)	0.61655 (13)	0.0406 (3)	
O1	0.5280 (4)	−0.00179 (13)	0.2404 (4)	0.0585 (10)	
O4	0.6120 (3)	−0.20100 (12)	−0.1933 (3)	0.0375 (7)	
H4	0.5719	−0.1881	−0.2794	0.056*	
N1	0.4855 (4)	−0.08217 (14)	0.3536 (4)	0.0380 (9)	
N2	0.4767 (4)	−0.15870 (15)	0.5130 (4)	0.0376 (9)	
C1	0.2725 (5)	−0.2232 (2)	0.4575 (6)	0.0498 (12)	
H1A	0.1688	−0.2180	0.4190	0.075*	
H1B	0.3083	−0.2233	0.5668	0.075*	
H1C	0.2955	−0.2589	0.4216	0.075*	
C2	0.6896 (5)	−0.02716 (17)	0.4936 (5)	0.0395 (11)	
C3	0.5627 (6)	−0.03336 (18)	0.3500 (5)	0.0430 (12)	
C5	0.3714 (5)	−0.1052 (2)	0.2168 (5)	0.0434 (12)	
H5	0.3047	−0.0741	0.1666	0.052*	
C6	0.2857 (5)	−0.1505 (2)	0.2668 (5)	0.0455 (12)	
C7	0.3424 (5)	−0.17558 (19)	0.4030 (5)	0.0406 (11)	
C9	0.5358 (5)	−0.11320 (17)	0.4837 (5)	0.0348 (10)	
C10	0.4374 (5)	−0.12880 (17)	0.1052 (5)	0.0374 (10)	
C11	0.5353 (5)	−0.17425 (18)	0.1479 (5)	0.0379 (10)	
H11	0.5612	−0.1893	0.2456	0.045*	
C12	0.5942 (5)	−0.19733 (17)	0.0490 (5)	0.0348 (10)	
H12	0.6610	−0.2270	0.0803	0.042*	
C13	0.5535 (5)	−0.17610 (17)	−0.0963 (5)	0.0347 (10)	
C14	0.4570 (5)	−0.13072 (17)	−0.1407 (5)	0.0387 (11)	
H14	0.4306	−0.1158	−0.2386	0.046*	
C15	0.4003 (6)	−0.1078 (2)	−0.0394 (5)	0.0466 (12)	
H15	0.3356	−0.0774	−0.0699	0.056*	
C16	0.7832 (6)	0.01649 (18)	0.5097 (6)	0.0474 (13)	
H16	0.7560	0.0414	0.4274	0.057*	
C17	0.9172 (6)	0.03188 (19)	0.6303 (6)	0.0490 (13)	
C18	0.9740 (6)	0.0014 (2)	0.7658 (6)	0.0521 (13)	
H18	0.9223	−0.0296	0.7817	0.063*	
C19	1.1042 (6)	0.0158 (2)	0.8764 (7)	0.0606 (15)	
H19	1.1399	−0.0052	0.9660	0.073*	
C20	1.1822 (6)	0.0617 (3)	0.8549 (8)	0.0702 (18)	
H20	1.2711	0.0711	0.9295	0.084*	
C21	1.1297 (7)	0.0930 (2)	0.7252 (8)	0.0658 (17)	
H21	1.1810	0.1246	0.7121	0.079*	
C22	1.0006 (6)	0.0778 (2)	0.6137 (7)	0.0571 (15)	
O2A	0.0777 (8)	−0.2032 (3)	0.1381 (8)	0.0398 (15)	0.568 (5)
O3A	0.1029 (9)	−0.1203 (3)	0.0273 (9)	0.0478 (18)	0.568 (5)
C8A	0.1483 (11)	−0.1543 (4)	0.1338 (13)	0.0344 (18)	0.568 (5)
C4A	−0.0570 (9)	−0.2119 (4)	0.0135 (11)	0.055 (2)	0.568 (5)
H4A1	−0.0980	−0.2479	0.0256	0.083*	0.568 (5)
H4A2	−0.0397	−0.2118	−0.0805	0.083*	0.568 (5)
H4A3	−0.1237	−0.1818	0.0119	0.083*	0.568 (5)
O2B	0.0944 (12)	−0.1436 (4)	0.0420 (13)	0.0398 (15)	0.432 (5)
O3B	0.0726 (12)	−0.2187 (4)	0.1889 (12)	0.0478 (18)	0.432 (5)
C8B	0.1406 (17)	−0.1798 (6)	0.1675 (17)	0.0344 (18)	0.432 (5)
C4B	−0.0390 (12)	−0.1602 (5)	−0.0732 (13)	0.055 (2)	0.432 (5)
H4B1	−0.0667	−0.1323	−0.1532	0.083*	0.432 (5)
H4B2	−0.1135	−0.1629	−0.0302	0.083*	0.432 (5)
H4B3	−0.0269	−0.1966	−0.1139	0.083*	0.432 (5)

### Atomic displacement parameters (Å^2^)

**Table:**

	*U*^11^	*U*^22^	*U*^33^	*U*^12^	*U*^13^	*U*^23^
Br1	0.0943 (5)	0.0404 (3)	0.1033 (6)	−0.0081 (3)	0.0720 (4)	−0.0062 (3)
S1	0.0530 (7)	0.0361 (6)	0.0348 (6)	−0.0079 (5)	0.0185 (5)	−0.0037 (5)
O1	0.089 (3)	0.0330 (17)	0.056 (2)	0.0130 (18)	0.029 (2)	0.0096 (17)
O4	0.0459 (19)	0.0332 (16)	0.0337 (16)	0.0035 (13)	0.0147 (15)	−0.0014 (13)
N1	0.050 (2)	0.0355 (19)	0.030 (2)	0.0093 (17)	0.0166 (17)	−0.0013 (16)
N2	0.044 (2)	0.041 (2)	0.0301 (19)	−0.0043 (18)	0.0160 (17)	−0.0071 (16)
C1	0.043 (3)	0.051 (3)	0.060 (3)	−0.008 (2)	0.025 (2)	−0.016 (3)
C2	0.061 (3)	0.026 (2)	0.041 (3)	0.004 (2)	0.031 (2)	−0.0029 (19)
C3	0.066 (3)	0.023 (2)	0.049 (3)	0.011 (2)	0.031 (3)	−0.002 (2)
C5	0.049 (3)	0.041 (2)	0.036 (3)	0.015 (2)	0.010 (2)	0.000 (2)
C6	0.038 (3)	0.054 (3)	0.043 (3)	0.009 (2)	0.013 (2)	−0.017 (2)
C7	0.043 (3)	0.040 (2)	0.045 (3)	0.004 (2)	0.023 (2)	−0.010 (2)
C9	0.044 (3)	0.034 (2)	0.030 (2)	0.006 (2)	0.019 (2)	−0.0039 (19)
C10	0.045 (3)	0.031 (2)	0.033 (2)	0.0082 (19)	0.011 (2)	−0.0016 (18)
C11	0.048 (3)	0.037 (2)	0.028 (2)	0.007 (2)	0.012 (2)	0.0058 (19)
C12	0.041 (3)	0.029 (2)	0.035 (2)	0.0046 (19)	0.014 (2)	0.0005 (18)
C13	0.042 (3)	0.030 (2)	0.033 (2)	−0.0039 (19)	0.016 (2)	−0.0048 (18)
C14	0.052 (3)	0.034 (2)	0.029 (2)	0.005 (2)	0.014 (2)	0.0027 (19)
C15	0.064 (3)	0.041 (2)	0.031 (2)	0.021 (2)	0.012 (2)	0.004 (2)
C16	0.069 (4)	0.024 (2)	0.061 (3)	0.004 (2)	0.038 (3)	−0.005 (2)
C17	0.058 (3)	0.030 (2)	0.071 (4)	−0.003 (2)	0.038 (3)	−0.015 (2)
C18	0.062 (3)	0.037 (3)	0.063 (3)	−0.010 (2)	0.030 (3)	−0.016 (3)
C19	0.066 (4)	0.052 (3)	0.063 (4)	0.004 (3)	0.023 (3)	−0.014 (3)
C20	0.055 (4)	0.071 (4)	0.091 (5)	−0.017 (3)	0.035 (3)	−0.050 (4)
C21	0.081 (5)	0.049 (3)	0.092 (5)	−0.014 (3)	0.060 (4)	−0.031 (3)
C22	0.069 (4)	0.037 (3)	0.086 (4)	−0.008 (3)	0.053 (3)	−0.023 (3)
O2A	0.031 (3)	0.038 (4)	0.048 (3)	−0.006 (3)	0.012 (3)	0.003 (3)
O3A	0.055 (4)	0.027 (3)	0.055 (4)	−0.004 (3)	0.013 (3)	0.002 (3)
C8A	0.037 (4)	0.019 (5)	0.045 (5)	0.015 (5)	0.013 (4)	0.001 (5)
C4A	0.036 (4)	0.057 (4)	0.063 (5)	0.000 (3)	0.006 (4)	0.013 (4)
O2B	0.031 (3)	0.038 (4)	0.048 (3)	−0.006 (3)	0.012 (3)	0.003 (3)
O3B	0.055 (4)	0.027 (3)	0.055 (4)	−0.004 (3)	0.013 (3)	0.002 (3)
C8B	0.037 (4)	0.019 (5)	0.045 (5)	0.015 (5)	0.013 (4)	0.001 (5)
C4B	0.036 (4)	0.057 (4)	0.063 (5)	0.000 (3)	0.006 (4)	0.013 (4)

### Geometric parameters (Å, º)

**Table:**

Br1—C22	1.915 (6)	C13—C14	1.387 (6)
S1—C2	1.747 (4)	C14—C15	1.377 (7)
S1—C9	1.753 (5)	C14—H14	0.9300
O1—C3	1.215 (5)	C15—H15	0.9300
O4—C13	1.374 (5)	C16—C17	1.441 (7)
O4—H4	0.8200	C16—H16	0.9300
N1—C9	1.355 (5)	C17—C18	1.392 (7)
N1—C3	1.382 (6)	C17—C22	1.397 (7)
N1—C5	1.472 (6)	C18—C19	1.370 (7)
N2—C9	1.293 (5)	C18—H18	0.9300
N2—C7	1.410 (6)	C19—C20	1.378 (8)
C1—C7	1.497 (6)	C19—H19	0.9300
C1—H1A	0.9600	C20—C21	1.357 (9)
C1—H1B	0.9600	C20—H20	0.9300
C1—H1C	0.9600	C21—C22	1.371 (8)
C2—C16	1.349 (6)	C21—H21	0.9300
C2—C3	1.479 (7)	O2A—C8A	1.348 (11)
C5—C10	1.527 (6)	O2A—C4A	1.432 (11)
C5—C6	1.534 (7)	O3A—C8A	1.232 (12)
C5—H5	0.9800	C4A—H4A1	0.9600
C6—C7	1.336 (7)	C4A—H4A2	0.9600
C6—C8A	1.477 (12)	C4A—H4A3	0.9600
C6—C8B	1.556 (17)	O2B—C8B	1.391 (17)
C10—C15	1.367 (6)	O2B—C4B	1.423 (15)
C10—C11	1.396 (6)	O3B—C8B	1.192 (17)
C11—C12	1.374 (6)	C4B—H4B1	0.9600
C11—H11	0.9300	C4B—H4B2	0.9600
C12—C13	1.373 (6)	C4B—H4B3	0.9600
C12—H12	0.9300		
			
C2—S1—C9	91.4 (2)	C15—C14—C13	119.7 (4)
C13—O4—H4	109.5	C15—C14—H14	120.1
C9—N1—C3	116.3 (4)	C13—C14—H14	120.1
C9—N1—C5	120.5 (4)	C10—C15—C14	121.3 (4)
C3—N1—C5	122.4 (4)	C10—C15—H15	119.3
C9—N2—C7	116.8 (4)	C14—C15—H15	119.3
C7—C1—H1A	109.5	C2—C16—C17	132.3 (5)
C7—C1—H1B	109.5	C2—C16—H16	113.9
H1A—C1—H1B	109.5	C17—C16—H16	113.9
C7—C1—H1C	109.5	C18—C17—C22	116.2 (5)
H1A—C1—H1C	109.5	C18—C17—C16	122.8 (5)
H1B—C1—H1C	109.5	C22—C17—C16	120.9 (5)
C16—C2—C3	119.6 (4)	C19—C18—C17	121.6 (5)
C16—C2—S1	130.3 (4)	C19—C18—H18	119.2
C3—C2—S1	110.0 (3)	C17—C18—H18	119.2
O1—C3—N1	122.6 (5)	C18—C19—C20	120.0 (6)
O1—C3—C2	127.0 (4)	C18—C19—H19	120.0
N1—C3—C2	110.5 (4)	C20—C19—H19	120.0
N1—C5—C10	110.8 (4)	C21—C20—C19	120.3 (6)
N1—C5—C6	108.5 (4)	C21—C20—H20	119.9
C10—C5—C6	112.2 (4)	C19—C20—H20	119.9
N1—C5—H5	108.4	C20—C21—C22	119.5 (6)
C10—C5—H5	108.4	C20—C21—H21	120.2
C6—C5—H5	108.4	C22—C21—H21	120.3
C7—C6—C8A	136.4 (6)	C21—C22—C17	122.4 (6)
C7—C6—C5	121.0 (4)	C21—C22—Br1	116.3 (4)
C8A—C6—C5	102.7 (5)	C17—C22—Br1	121.3 (4)
C7—C6—C8B	110.8 (7)	C8A—O2A—C4A	115.6 (8)
C5—C6—C8B	127.6 (7)	O3A—C8A—O2A	122.4 (10)
C6—C7—N2	122.4 (4)	O3A—C8A—C6	127.0 (9)
C6—C7—C1	125.0 (5)	O2A—C8A—C6	110.5 (8)
N2—C7—C1	112.6 (4)	O2A—C4A—H4A1	109.5
N2—C9—N1	126.2 (4)	O2A—C4A—H4A2	109.5
N2—C9—S1	122.0 (3)	H4A1—C4A—H4A2	109.5
N1—C9—S1	111.8 (3)	O2A—C4A—H4A3	109.5
C15—C10—C11	118.0 (4)	H4A1—C4A—H4A3	109.5
C15—C10—C5	121.9 (4)	H4A2—C4A—H4A3	109.5
C11—C10—C5	120.1 (4)	C8B—O2B—C4B	114.1 (10)
C12—C11—C10	121.5 (4)	O3B—C8B—O2B	125.5 (14)
C12—C11—H11	119.2	O3B—C8B—C6	133.4 (12)
C10—C11—H11	119.2	O2B—C8B—C6	100.8 (10)
C13—C12—C11	119.3 (4)	O2B—C4B—H4B1	109.5
C13—C12—H12	120.3	O2B—C4B—H4B2	109.5
C11—C12—H12	120.3	H4B1—C4B—H4B2	109.5
C12—C13—O4	117.8 (4)	O2B—C4B—H4B3	109.5
C12—C13—C14	120.0 (4)	H4B1—C4B—H4B3	109.5
O4—C13—C14	122.2 (4)	H4B2—C4B—H4B3	109.5

### Hydrogen-bond geometry (Å, º)

Cg1 is the centroid of the C10–C15 benzene ring.

**Table:**

*D*—H···*A*	*D*—H	H···*A*	*D*···*A*	*D*—H···*A*
O4—H4···N2^i^	0.82	1.96	2.782 (4)	178
C4*B*—H4*B*1···S1^ii^	0.96	2.80	3.621 (12)	144
C1—H1*C*···*Cg*1^iii^	0.96	2.78	3.585 (5)	142

Symmetry codes: (i) *x*, *y*, *z*−1; (ii) *x*−1, *y*, *z*−1; (iii) *x*−1, −*y*−1/2, *z*−3/2.
